# Supplementary material for: FathomNet: A global image database for enabling artificial intelligence in the ocean
Source: Sci Rep. 2022 Sep 23;12:15914. doi: 10.1038/s41598-022-19939-2 (PMC9508077; doi:10.1038/s41598-022-19939-2)
Supplement: Supplementary file 1 — Supplementary Information. [file 41598_2022_19939_MOESM1_ESM.pdf]

Supplementary Information for *FathomNet: A global image database for enabling artificial intelligence in the ocean*

Kakani Katija[1,2,3,*], Eric Orenstein[1], Brian Schlining[1], Lonny Lundsten[1], Kevin Barnard[1], Giovanna Sainz[1], Oceane Boulais[4], Megan Cromwell[5], Erin Butler[6], Benjamin Woodward[6], and Katy Croff Bell[7]

[1]Monterey Bay Aquarium Research Institute, Research and Development, Moss Landing, 95039, USA
[2]California Institute of Technology, Graduate Aerospace Laboratories, Pasadena, 91125, USA
[3]Smithsonian Institution, National Museum of Natural History, Washington DC, 37012, USA
[4]NOAA, Southeast Fisheries Science Center, Key Biscayne, 33149, USA
[5]NOAA, National Centers for Environmental Information, Stennis Space Center, 39529, USA
[6]CVision AI Inc., Research and Development, Medford, 02155, USA
[7]Ocean Discovery League, Saunderstown, 02874, USA
[*]kakani@mbari.org

Supplementary Table

Table S1. Cost comparison between ImageNet, COCO, and FathomNet. These values should be understood as estimates since most papers do not explicitly state the number of crowd worker hours nor compensation rates. $*$ = values estimated from Deng et al.[1] $\star$ = values estimated from Lin et al.[2] † = MBARI contributions only. $\diamond$ = rate drawn from Sorokin and Forsyth.[3] AMT = Amazon Mechanical Turk. MBARI VL = MBARI Video Laboratory.

Dataset	Ann. source	Ann. hours	Cost per hour	Est. total cost
ImageNet-category labels*	AMT	75,755	3.25^{\diamond}$	$246,203
COCO-category labels*	AMT	17,751	3.25^{\diamond}$	$57,691
COCO-instance spotting*	AMT	8,417	3.25^{\diamond}$	$27,355
FathomNet-benthic†	MBARI VL	1651	$80.00	$132,080
FathomNet-midwater†	MBARI VL	412	$80.00	$32,960

Supplementary Figure

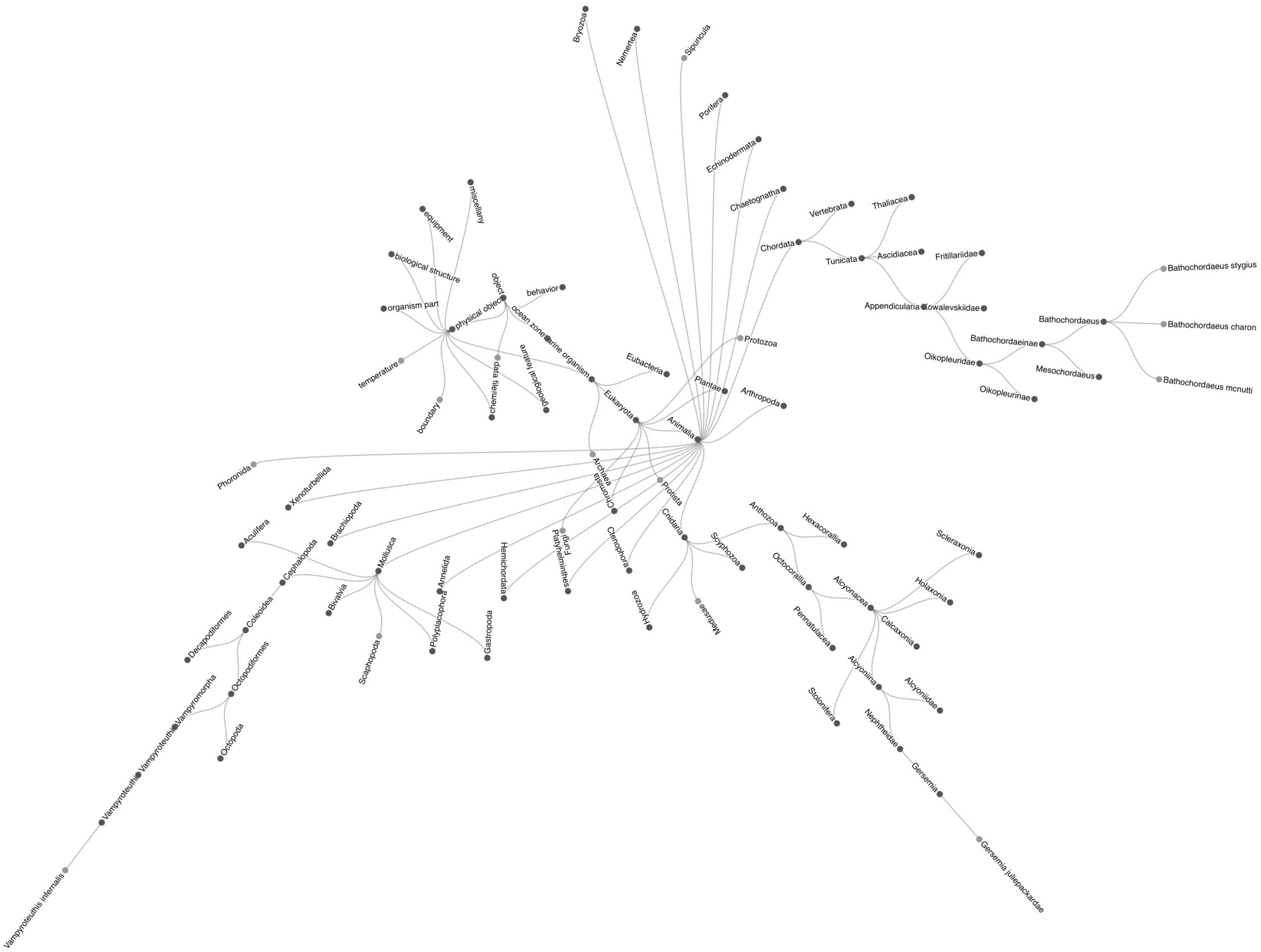

Figure S1. FathomNet's concept tree is currently built based on MBARI's VARS database. In addition to marine organisms and their taxonomic hierarchies (object/marine organism; authors' favorites include the giant larvacean *Bathochordaeus mcnutti*, octocoral *Gersemia juliepackardae*, and the cephalopod *Vampyroteuthis infernalis*), the FathomNet concept tree also includes concepts for trash (object/physical object/miscellany/trash) and geologic features (object/physical object/geologic feature).

Captions for Supplementary Video

Video S1. Object detector (trained on MBARI image data) performance on video collected by NOAA's ROV *Deep Discoverer* during a midwater transect.

References

1. Deng, J. *et al.* ImageNet: A large-scale hierarchical image database. In *2009 IEEE Conference on Computer Vision and Pattern Recognition*, 248–255, DOI: 10.1109/CVPR.2009.5206848 (2009).
2. Lin, T.-Y. *et al.* Microsoft coco: Common objects in context. In *European conference on computer vision*, 740–755 (Springer, 2014).
3. Sorokin, A. & Forsyth, D. Utility data annotation with amazon mechanical turk. In *2008 IEEE computer society conference on computer vision and pattern recognition workshops*, 1–8 (IEEE, 2008).
